# The role of the endocannabinoid system in the interplay of adverse childhood experiences and interleukin 6 in individuals with borderline personality disorder

**DOI:** 10.1007/s00213-025-06809-8

**Published:** 2025-05-17

**Authors:** Jennifer Spohrs, Valentin Kühnle, Stefan O. Reber, David Mikusky, Niklas Sanhüter, Ana Macchia, Sandra Nickel, Birgit Abler

**Affiliations:** 1 Department for Child and Adolescent Psychiatry and Psychotherapy, Ulm University Medical Centre, Ulm, Germany; 2 Clinic for Psychiatry, Psychotherapy and Psychotraumatology, Military Medical Centre, Ulm, Germany; 3 Department of Psychiatry and Psychotherapy III, Ulm University Medical Centre, Ulm, Germany; 4 Laboratory for Molecular Psychosomatics, Department of Psychosomatic Medicine and Psychotherapy, Ulm University Medical Center, Ulm, Germany

**Keywords:** Adverse childhood experiences, Endocannabinoid system, Interleukin 6, Anandamide, 2-Arachidonoylglycerol, Suicidal behaviour, Non-suicidal self-injury

## Abstract

**Rationale:**

Adverse childhood experiences (ACEs) have been identified as a major risk factor for psychiatric disorders from childhood to adult life along with the dysregulation of neuroendocrinological processes mediating stress and inflammation. The endocannabinoid system (ECS) has been found to play a putative role in the release of inflammatory cytokines.

**Objective:**

We investigated the role of the ECS in the interplay between ACEs and interleukin 6 (IL-6) as an inflammatory marker.

**Methods:**

We analysed ACEs (CTQ, Bernstein et al. 2003), plasma IL-6 and endocannabinoid concentrations (anandamide (AEA) and 2-arachidonoylglycerol (2-AG) in a cohort comprising 48 female individuals diagnosed with borderline personality disorder (BPD) and 31 matched healthy controls (HCs).

**Results:**

We found higher IL-6 levels in individuals with BPD compared to HCs and, across all study participants, observed significant positive correlations between AEA, 2-AG and IL-6 levels. CTQ sum scores correlated positively with IL-6 concentrations at a trend level (statistically significant for sexual abuse). Correlations between CTQ sum scores and IL-6 levels were particularly strong in participants with low endocannabinoid levels (lowest three quartiles; *n* = 57) while in the quartile with the highest endocannabinoid levels (*n* = 19), no correlations were evident. Furthermore, an exploratory analysis applying a median split for IL-6 levels revealed that the number of individuals with recent suicide attempts (< 1 month ago) was significantly higher in the high IL-6 levels group (OR = 0.22; 95%CI = 0.06–0.86).

**Conclusion:**

Our findings support the bidirectional link between ACEs and immune system alterations and suggest that endocannabinoids may counteract the stress-inflammatory response.

## Introduction

Adverse childhood experiences (ACEs) have been identified as major risk factors for psychiatric disorders (Gilbert et al. 2009; Danese and Baldwin 2017), emotional and behavioural difficulties (Suliman et al. 2009; Kessler et al. 2010), functional impairment (Copeland et al. 2018) and poorer treatment response (Nanni et al. 2012) in a dose dependant manner. These ACEs may be sexual, physical or psychological abuse or neglect (physical or emotional) (Gilbert et al. 2009). They are highly prevalent, with more than 60% of children and adolescents experiencing a potentially traumatic event before the age of 16 (McLaughlin et al. 2013).

Common psychiatric disorders that have been linked to ACEs include psychotic disorder (Arseneault et al. 2011; Varese et al. 2012), depression (Kessler 1997), anxiety and substance disorder (Copeland et al. 2018), bipolar disorder (Agnew-Blais and Danese 2016), substance use disorder (Kirsch et al. 2020), posttraumatic stress disorder (PTSD) (Cloitre et al. 2009; McLaughlin et al. 2013) and borderline personality disorder (BPD). Hereby, patients with BPD have been found to be more than 13-fold more likely to report ACEs than non-clinical populations (Porter et al. 2020) with neurobiological factors potentially moderating this link. Wilson et al. (2021) report that childhood maltreatment may modify genes relevant for hypothalamic-pituitary-adrenals (HPA) axis control, which has been linked to subsequent vulnerability to BPD and may play a direct or indirect causal role in the pathways underlying heightened inflammation levels (Heim 2000; Danese and J Lewis 2017). The fact, that ACEs can alter the development and regulation of the HPA axis from childhood to adult life has already been indicated by early research (Levine et al. 1957) and understanding the link between ACEs and the neuroendocrine and neuroinflammatory stress response may aid in elucidating their clinical effects.

Heightened inflammatory reactivity has also been linked to childhood trauma, such as abuse and neglect, a parameter found to contribute to both physical and psychological pathology, for example heightened stress responses, sleep disturbances, substance abuse, obesity and mental health (Gilbert et al. 2009; Beilharz et al. 2020). In a longitudinal study, Danese et al. (2007) demonstrated that childhood maltreatment correlated with inflammation levels at age 32, which followed a dose-dependent manner depending on the severity of maltreatment, a finding, that has since been replicated in various studies (Coelho et al. 2014; Baumeister et al. 2016). These heightened inflammatory responses have also been observed in relation to daily stressors in people who reported childhood maltreatment (Fagundes et al. 2013). Extending its impact on biological processes, childhood trauma may also affect sleep, diet and emotion regulation, which may indirectly modulate inflammation (Danese and Tan 2014; Gregory and Sadeh 2016). Moreover, children experiencing ACEs may be more vulnerable to infections (Cohen et al. 1991), which, in turn, may contribute to inflammatory processes.

While increased levels have been reported for various inflammatory markers in the context of ACEs, interleukin 6 (IL-6) has been particularly consistently reported as increased in adults with a history of ACEs (Danese et al. 2007; Baumeister et al. 2016; Heard-Garris et al. 2020). In addition, during a social stress test, individuals with ACEs exhibited a stronger increase in IL-6 (Carpenter et al. 2010). Strikingly, in their meta-analysis, Neupane et al. (2023) found that suicidal behaviour, which is also prominent in BPD (Ducasse et al. 2020; Walker et al. 2022), was particularly associated with higher IL-6 levels among other immune-related biomarkers. The link between IL-6 levels, suicidal behaviour and childhood trauma has also been studied by Walker et al. (2022), who reported a significant association between suicidal ideation and inflammation, although childhood maltreatment did not play a mediating role. However, in their meta-analysis, Baumeister et al. (2016) found that childhood maltreatment, which is associated with greater suicide risk (Bebbington et al. 2009), has an impact on inflammation.

As another player in inflammatory processes, the endocannabinoid system (ECS) has become a central topic in research. On the behavioural level, the endocannabinoids anandamide (AEA) and 2-arachinoylglycerol (2-AG) have been found to exert an anxiolytic effect (Lutz et al. 2015) and cannabis or cannabidiol are thus commonly used for self-medication in BPD (Vest et al. 2018). Additionally, AEA promotes extinction learning in fear processes (Spohrs et al. 2021) and AEA as well as 2-AG have been found to have anti-inflammatory effects (Giacobbe et al. 2021). Previous studies have shown that increased endocannabinoid signalling inhibits the release of pro-inflammatory cytokines and enhances the release of anti-inflammatory cytokines (Hill et al. 2018; Henshaw et al. 2021). There are certainly many cellular processes involved in the regulation of immunological pathways, with the ECS potentially functioning as a modulator of these processes.

Research on ACEs and the ECS remains scarce. However, Marusak et al. (2024) have found higher AEA levels to be associated with PTSD symptom severity in an adolescent sample and Mazurka et al. (2024) found elevated levels of 2-AG, but only in patients with major depressive disorder who reported childhood maltreatment. Higher endocannabinoid concentrations have also been found in the hair of postpartum mothers and their children with childhood maltreatment (Koenig et al. 2018). Adding to the literature, we found higher AEA levels and, by trend higher 2-AG levels in individuals with BPD and an effect of genotype (FAAH_rs324420) was associated with higher levels of depression (Spohrs et al. 2023). In contrast, (Wingenfeld et al. 2018) found reduced AEA in hair samples in a pilot study with BPD patients. To shed light on the described mechanisms, our hypothesis 1 investigated whether IL-6, as an inflammatory parameter, was elevated in a sample of individuals with BPD, all of whom presented high levels of ACEs, compared to healthy controls, as suggested by previous studies (MacDowell et al. 2020). Hypothesis 2 investigated whether childhood trauma as assessed with the Childhood Trauma Questionnaire (CTQ (Bernstein et al. 2003) impacted these levels and if the endocannabinoids AEA and 2-AG modulated these effects (hypothesis 3). Lastly, in an exploratory manner, we assessed the link between self-harm and suicidal behaviour on IL-6, as suggested in previous research in individuals experiencing ACEs (4) (Bebbington et al. 2009).

## Methods

The study was approved by the Ethics Committee of Ulm University, Germany (#221/21) and conducted in accordance with the guidelines of the Declaration of Helsinki. All participants, patients and healthy controls, provided informed consent before inclusion to the study.

### Participants

Participation in the study was offered to all 73 patients who took part in the 8-week dialectical behaviour therapy (DBT) inpatient program for BPD at the Department of Psychiatry and Psychotherapy III of Ulm University Hospital between July 2021 and July 2022. 54 individuals with a BPD diagnosis gave their consent to participate in the study. Data on endocannabinoid levels from a largely overlapping sample including diagnostic procedures and comorbidities can be found in Spohrs et al. (2023). All individuals included in the BPD group (*n* = 48) fulfilled the diagnostic criteria for BPD. Among them, 19 participants also met criteria for posttraumatic stress disorder (PTSD) and 34 were diagnosed with a current first or recurring major depressive episode. Regarding psychopharmacological treatment, in the BPD group, 40 participants were taking antidepressants at the time of the study, 11 were receiving a low dose of neuroleptic medication mainly to facilitate sleep and 4 were prescribed methylphenidate. In terms of substance use history, 8 participants reported lifetime alcohol abuse, 4 lifetime dependency and 3 reported active use within the past 6 months. A total of 7 participants reported lifetime abuse and 2 dependency of illicit drugs, although none reported abuse or dependency in the past 6 months. Lifetime cannabis abuse was reported by 12 individuals, with one reporting use in the past 6 months. 23 individuals (47.9%) with BPD were regular smokers, consuming on average 7,2 cigarettes per day.

Healthy control subjects (HCs) with no history of psychiatric disorder including substance use disorders were matched to individuals with BPD based on their gender, age, body mass index (BMI) and nicotine use. 15 HC (48.4%) were smokers and consumed on average 4.9 cigarettes per day. We present data from 31 HCs and the 48 individuals with BPD of the total sample of 54 individuals with BPD from which reliable IL-6 data could be obtained. IL-6 levels could not be assessed from blood samples from 5 individuals with BPD and 1 HC; data from 1 patient with indication of an infectious process and elevated C-reactive protein (CRP) levels were excluded. Failure to assess IL-6 levels in 6 subjects, along with the exclusion of data from the subject with elevated CRP, accounts for the discrepancies observed between this sample and that presented in Spohrs et al. (2023) regarding differences in endocannabinoid levels between patients and controls. Characteristics of the sample included are shown in Table 1.

### Blood sampling

Blood samples were collected under fasting conditions in the morning from all participants. Blood samples from individuals with BPD were obtained at up to three time points when blood was drawn for clinical routine, i.e. upon admission, in the middle of the program (after approximately 4 to 5 weeks) and upon discharge (after approximately 7 to 10 weeks). In HCs, only one blood sample was obtained.

In this paper, we present data from blood samples obtained upon admission. We measured plasma levels of IL-6 (Human IL-6 Quantikine HS ELISA Kit, R&D Systems Europe, Ltd.; lowest standard 0.16 pg/ml), according to the manufacturers’ instructions, as well as AEA and 2-AG by mass spectrometry analysis following blood-to-plasma processing. Extraction and analysis of plasma endocannabinoids were carried out according to previously described protocols (Spohrs et al. 2021).

### Questionnaires and assessment of self harm

BPD symptom severity upon admission in the patients was measured using the short version of the Borderline Symptom List (BSL-23)(Bohus et al. 2009). This self-rating instrument, with good psychometric properties, consists of 23 statements, with which patients rate their agreement using a 5-point Likert scale ranging from „not at all“ to „very much“. Here, we analysed the mean score on the BSL-23 as the patient-reported outcome. Depressive symptoms were measured using the German version of the Beck Depression Inventory, second edition (BDI-II, German version, (Hautzinger et al. 2009). The Childhood Trauma Questionnaire (CTQ) (Bernstein et al. 2003) assesses childhood trauma on five dimensions: emotional, sexual, and physical abuse, and emotional and physical neglect, by means of 25 items, which can be rated on a five-point Likert scale. Frequency of self-harm (self-injury, suicidal ideation and suicide attempts) was assessed via interview along with the self-rating questionnaires. In HC subjects, BSL-23, BDI-II and CTQ scores were obtained directly before or after the blood draw. Regarding self-harm, the last episode of NSSI and the last suicide attempt were inquired. Suicidal ideation was assessed over the past month. Individuals with BPD reported the average number of days per week with suicidal thoughts during the past month.

### Statistics

Calculations were performed using Microsoft Excel and Statistica for Windows. Comparisons between groups (individuals with BPD /controls) were conducted using Chi-square (X²) tests, independent sample t-tests (two-tailed) or Mann-Whitney U-test as appropriate. Pearson’s correlations were calculated to investigate the relationship between IL-6, endocannabinoid serum levels and psychopathology. Odds ratios were calculated to compare individuals with BPD with low vs. high IL-6 levels regarding recent self-injury or suicide attempts. Given the small sample size, we present both the results for the full sample and those obtained after outlier correction. Outliers were identified using z-score analysis, with values exceeding 2 standard deviations above group mean being excluded from analysis.

**Table 1:** Demographic variables, questionnaire results, IL-6 and endocannabinoid levels

	Individuals with BPD (*N* = 48)	Healthy controls (*N* = 31)	Group differences
Gender	41 females (85.4%)	26 females (85.4%)	X²=0.03, *p* = 0.85^b^
Age (M ± SD)	27.12 ± 10.13 [18–61 y]	25.03 ± 3.75 [18–35 y]	*t*(65) = 1.30, *p* = 0.20^a^
Body Mass Index: kg/m² (M ± SD)	28.57 ± 8.17	26.84 ± 6.98	*t*(73) = 1.00, *p* = 0.32^a^
BDI-II sum score (M ± SD)	37.37 ± 8.48	5.06 ± 3.34	*t*(55) = 22.11, *p* < 0.001^a^
BSL-23 sum score (M ± SD)	50.52 ± 16.03	2.87 ± 2.40	*t*(75) = 19.4, *p* < 0.001^a^
CTQ sum score (M ± SD)	61.38 ± 16.72	32.83 ± 6.15	*t*(67) = 10.93, *p* < 0.001^a^
CTQ emotional abuse	16.86 ± 6.11	7.06 ± 2.26	*t*(68) = 10.26, *p* < 0.001^a^
CTQ physical abuse (M ± SD)	9.04 ± 4.33	5.53 ± 1.06	*t*(58) = 5.45, *p* < 0.001^a^
CTQ sexual abuse (M ± SD)	10.06 ± 6.35	5.70 ± 2.40	*t*(68) = 4.36, *p* < 0.001^a^
CTQ emotional neglect	15.28 ± 6.22	7.22 ± 3.00	*t*(75) = 7.80, *p* < 0.001^a^
CTQ physical neglect	10.14 ± 3.13	6.29 ± 1.92	*t*(79) = 6.86, *p* < 0.001^a^
IL-6 levels, pmol/ml plasma (M ± SD)	1.49 ± 0.97	1.10 ± 1.02	*t*(62) = 1.72, *p* = 0.04^a+^
AEA levels, pmol/ml plasma (M ± SD)	0.99 ± 0.55	0.69 ± 0.30	*t*(75) = 3.08, *p* = 0.003^a^
2-AG levels, pmol/ml plasma (M ± SD)	1.49 ± 1.70	0.95 ± 0.56	*t*(61) = 2.02, *p =* 0.05^a^

*Notes*^a^ independent sample t-test; ^b^ chi-square-test; + one-tailed test; M: mean; SD: standard deviation; BDI-II: Beck’s Depression Inventory; BSL-23: Borderline Symptom List; CTQ: Childhood trauma questionnaire; IL-6: interleukin 6; 2-AG = 2-arachidonoylglycerol; AEA = Anandamide; n.s.=not significant; M = mean; SD = standard deviation; Body Mass Index: missing data in 1 patient; BDI: missing data in 7 individuals with BPD; BSL: missing data in 4 individuals with BPD; CTQ: missing data in 3 individuals with BPD

## Results

Individuals with BPD and HCs were successfully matched with no group differences regarding age, gender and body mass index (see Table 1).

As expected, individuals with BPD scored significantly higher in the questionnaires BDI-II, BSL and CTQ, compared to HCs. In line with previous literature (Kahl et al. 2006; Ogłodek et al. 2016), we found significantly higher plasma IL-6 levels in the individuals with BPD compared to HCs (one-tailed t-test, see Table 1). To further investigate these differences, we calculated correlations between endocannabinoid levels (reported in Spohrs et al. 2023) and IL-6 levels as well as IL-6 levels and psychopathology measures. Across all (BPD and HC) 79 study participants, we found significant positive correlations between both AEA and 2-AG with IL-6 levels (AEA: *r* = 0.31, *p* = 0.005; 2-AG: *r* = 0.24, *p* = 0.032) and a trend when analysing individuals with BPD only (positive correlation AEA - IL-6: *r* = 0.28, *p* = 0.052; 2-AG – IL-6: *r* = 0.27, *p* = 0.059). As expected, IL-6 levels correlated positively with the body mass index (*r* = 0.39, *p* < 0.001). The significant correlations with IL-6 levels presented here were more pronounced after correction for three outliers in IL-6 scores (values > 2 standard deviations above group mean in two individuals with BPD and one HC). On the other hand, after correction for one outlier in BDI-II scores, no correlations were found between psychometric measures (BSL and BDI-II) and IL-6 or endocannabinoid levels.

CTQ sum scores correlated with IL-6 levels by trend (*r* = 0.217; *p* = 0.060) and significantly (*r* = 0.392; *p* = 0.001) after correction for the three outliers. Regarding CTQ subscores, we found a significant positive correlation between IL-6 levels and “sexual abuse” (*r* = 0.24; *p* = 0.035; *r* = 0.449; *p* < 0.001 after outlier correction).

**Fig. 1:**
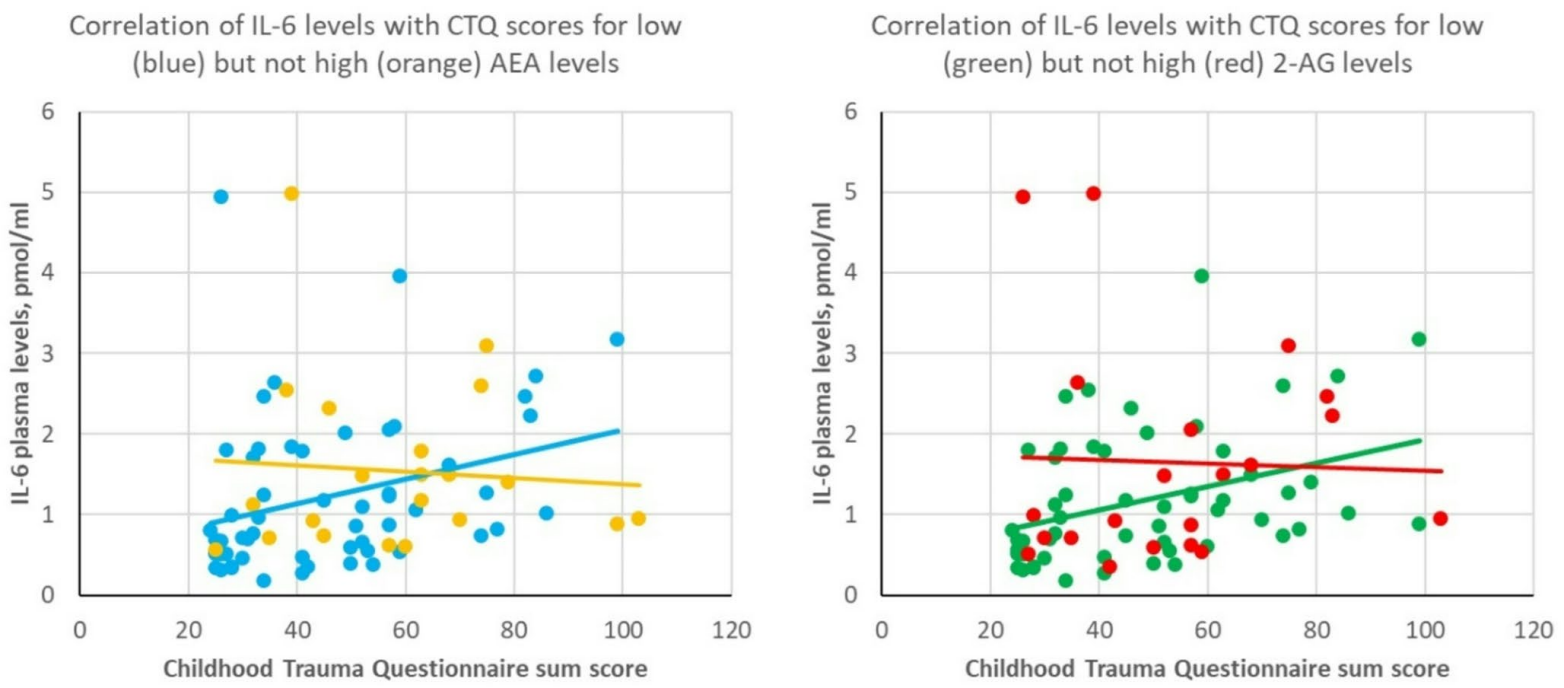
Correlations of IL-6 and CTQ in participants from the 3 lower quartiles of anandamide (AEA, left graph) and 2-arachidonoylglycerol (2-AG, right graph) levels (blue/green) and from the highest quartile (orange/red)

Based on previous findings of interindividual differences in AEA concentrations (Spohrs et al. 2021), we divided the whole group into quartiles. This approach was used to explore whether individuals with the highest AEA concentrations exhibit beneficial effects. We applied the same stratification to 2-AG to investigate whether the observed mechanisms are specific to AEA or reflect broader effects shared by both endocannabinoids. Interestingly, positive correlations between CTQ sum scores and IL-6 levels were particularly strong in participants with low AEA levels (lower three quartiles; *n* = 57) with correlations of *r* = 0.28; *p* = 0.0325 for CTQ sum scores (see Fig. 1) and *r* = 0.29; *p* = 0.011 for “sexual abuse”. In contrast, in the quartile with the highest AEA levels (*n* = 19), no correlations were evident (*r* < 0.1). Similarly and likely related to the positive correlation found between AEA and 2-AG levels (*r* = 0.62; *p* < 0.0001), we also revealed strong positive correlations between CTQ sum scores and IL-6 levels in participants with low 2-AG levels (3 lower quartiles; *n* = 55), with correlations of *r* = 0.36; *p* = 0.007 for CTQ sum scores (see Fig. 1), *r* = 0.39; *p* = 0.003 for “sexual abuse”, *r* = 0.31, *p* = 0.019 for “emotional neglect” and *r* = 0.27; *p* = 0.044 for “physical neglect”, while in the quartile with the highest 2-AG levels (*n* = 21), no correlations were evident (*r* < 0.1).

**Fig. 2:**
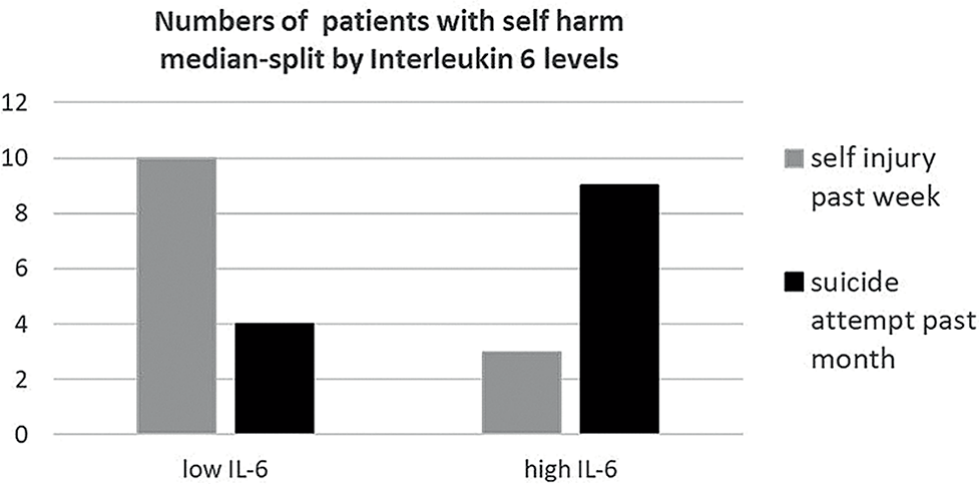
Odds ratios for numbers of individuals with BPD with self-injury in the past week and suicide attempts in the past month differ significantly between individuals with BPD with low or high (median-split) interleukin (IL-6) levels

To explore the relationship between IL-6 levels and recent suicide attempts, self-injury and suicidal ideation, we performed a median split of the patient group and compared individuals with BPD with low IL-6 levels to those with high levels. Given the comparable group sizes resulting from the median split, we were able to conduct further analysis to examine group differences. Regarding self-injury within the past week (data from 43 individuals with BPD available), 10 of 13 individuals with BPD (see Fig. 2) reporting self-injury within the past 7 days were in the low IL-6 level group indicating a significant difference (OR = 6.33; 95% CI = 1.48–27.18).

Suicide attempts within the past month among individuals with BPD with a history of suicide attempts (*n* = 28) were reported by 4 of 14 individuals with BPD in the low IL-6 level group and by 9 of 14 individuals with BPD in the high IL-6 levels group (see Fig. 2) indicating a significant difference (OR = 0.22; 95%CI = 0.06–0.86). Individuals with BPD in the low IL-6 group reported suicidal ideation at a mean of 3.10 days per week (SD = 2.38) while individuals with BPD in the high IL-6 group reported suicidal ideation at 4.75 days per week (mean; SD = 2.49) reflecting a significant difference (Mann-Whitney U-test: Z = 2.04; *p* = 0.041).

## Discussion

Based on previous findings demonstrating the involvement of ACEs in the neuroendocrine response throughout the lifespan, we investigated differences in IL-6 levels between individuals with BPD with BPD and HCs, as well as the links between ACEs, endocannabinoids and IL-6. In an exploratory analysis, we additionally examined the relationship between self-harm, suicidal ideation and IL-6 levels.

As hypothesised, we found higher IL-6 levels in the BPD group compared to the HCs. Across the entire sample, significant positive correlations were observed between AEA, 2-AG and IL-6. We also found a link between CTQ scores and IL-6 levels, with the *sexual abuse* subscale yielding a significant positive correlation. These correlations were particularly pronounced in the three quartiles with the lowest AEA and 2-AG concentrations, while no correlations were evident in the quartile with the highest AEA and 2-AG concentrations.

Lastly, our exploratory analysis, applying a median split on IL-6 values in the BPD group, revealed that more individuals with BPD with self-injurious behaviour within the past week were in the group with lower IL-6 levels, while a higher number of individuals with BPD with suicide attempts within the past month and also more frequent suicidal ideation was found in the high IL-6 group.

### Higher IL-6 in BPD compared to HCs

When comparing individuals with BPD and HCs, we found higher plasma IL-6 levels in individuals with BPD, which aligns well with previous research findings focussing on psychiatric samples (Pace et al. 2006; Hoge et al. 2009) as well as populations that experienced ACEs (for review see Chen et al., 2021). When examining CTQ scores, we observed a positive correlation with IL-6 levels by trend (significant after outlier correction) and a significant correlation for the subscale *sexual abuse*. This is in line with suggestions that “severe” traumatic experiences, such as physical and sexual abuse might have the greatest impact on inflammatory markers (for review see Brown et al. 2021, meta-analysis by Baumeister et al. 2016). However previous research findings on this topic remain mixed.

### ACEs, IL-6 and endocannabinoids

To further investigate the neuroendocrinological parameters, we analysed the link between endocannabinoids and IL-6. As previously reported, AEA and 2-AG concentrations were higher in individuals with BPD than in the HCs (Spohrs et al. 2023). Across the entire sample, we observed a positive correlation between AEA, 2-AG and IL-6, which in the BPD group was evident by trend. The observation of elevated endocannabinoids alongside increased IL-6 concentrations in the individuals with BPD may indicate that activation of the ECS was insufficient to effectively counteract inflammatory processes. To gain a more nuanced understanding of this relationship, we stratified the entire sample into quartiles, in line with the approach taken in our previous research on endocannabinoids (Spohrs et al. 2021), and found that the quartile with the highest AEA and also the partially overlapping quartile with the highest 2-AG levels showed no correlation between CTQ scores and IL-6 levels, whereas significant positive correlations were observed in the three lower AEA/2-AG quartiles for CTQ scores and IL-6 levels. These findings align with previous research on anti-inflammatory effects of endocannabinoids (Henshaw et al. 2021). This observation could be interpreted to indicate that compensatory activation of the ECS may not only exert the previously found anxiolytic effects (Lutz et al. 2015) but may also function as a potential regulator of stress-induced inflammation and stress-related homeostasis. This interpretation aligns with the findings of Kerr et al. (2012), who found that fatty acid amide hydrolase inhibition, the enzyme that degrades anandamide, led to a reduction in pro-inflammatory cytokines. However, this interpretation remains tentative and requires replication in larger samples.

### IL-6, self-harm and suicidal tendencies

Our exploratory analyses yielded interesting results. After performing a median split for IL-6, we found that in the lower IL-6 group, more individuals with BPD had engaged in self-harm in the past week, whereas more individuals with BPD in the high IL-6 group reported suicide attempts within the past month as well as a higher frequency of suicidal ideation with more days per week with suicidal thoughts. These findings align with previous results, who found elevated pro-inflammatory interleukins in individuals with suicidal behaviour (see meta-analysis by González-Castro et al. 2021). While non-suicidal self-injury (NSSI) is frequently reported to reduce subjective stress in individuals with BPD (Klonsky 2007; Edmondson et al. 2016), this distress regulation might explain the lower inflammatory markers in the following days. However, this interpretation requires further scientific examination. Supporting this, Kindler et al. (2022) did not find a significant correlation between IL-6 and self-harm in a sample of individuals engaging in NSSI.

Meanwhile, recent suicide attempts, and frequent suicidal thoughts can be interpreted as a manifestation of immense psychological strain, which might explain the greater numbers in the high IL-6 group, consistent with previous studies (Serafini et al. 2013; Neupane et al. 2023). However, these data must be cautiously interpreted, as the directionality of the observed associations remains speculative.

### Limitations

Our findings should be interpreted in light of several limitations. Firstly, the sample sizes were uneven and relatively small, with a high percentage of female participants. The small sample sizes might also explain, why no correlations between IL-6 and clinical questionnaires were found. Since the investigation of endocannabinoids in BPD is still in its early stages, we did not control for variations in the menstrual cycle. Additionally, our findings may have been influenced by psychopharmacological medication, particularly antidepressants, which were used by a large proportion of the BPD group at the time of the study.

Because of the small sample size and the partly exploratory nature of the study, we chose not to apply corrections for multiple comparisons in this analyses. After addressing IL-6 and CTQ differences in BPD and HC, our primary goal was to identify the role of the ECS and generate hypotheses for future research, rather than to make definitive claims based on statistical significance. Applying strict multiple comparisons adjustments in this context could increase the risk of Type II errors, potentially obscuring meaningful signals that warrant further investigation. However, therefore our findings have to be interpreted with the appropriate caution.

Furthermore, there is a broad spectrum of immune-related biomarkers, such as anti-inflammatory cytokines, that we were unable to include in this study. Despite these limitations, the present data provide initial insights into the interplay of ACEs, inflammation and endocannabinoids. These findings need to be replicated and assessed in combination with other parameters to gain a more comprehensive understanding of the underlying mechanisms.

### Future directions

Available psychopharmacological interventions present limited effectiveness in BPD, a patient group characterised by a wide array of symptoms. Modulation of the ECS and inflammatory processes may provide multiple benefits for this highly affected patient group. It may serve an anxiolytic effect on anxiety symptoms (Bergamaschi et al.), promote extinction learning during trauma confrontation (Rabinak and Phan 2014; Mayo et al. 2020; Spohrs et al. 2021), and regulate inflammatory processes, which are dysregulated in BPD and individuals with a higher load of ACEs. Findings from previous studies suggest that endocannabinoid modulation may offer beneficial effects as pharmaceutical add-ons or as specific treatments integrated into therapeutic interventions. However, more research in humans is necessary to better understand the underlying mechanisms and additional neuroendocrinological processes involved. Nevertheless, the results underscore the important role of the ECS in psychiatric and neuroendocrinological processes, highlighting its potential therapeutic benefits. Future research should investigate the effects of ECS modulation in larger samples to fully elucidate its role in therapeutic interventions.

## Data Availability

Data and material can be shared upon individual request to the authors.
